# Cecal Microbiota Modulates Fat Deposition in Muscovy Ducks

**DOI:** 10.3389/fvets.2021.609348

**Published:** 2021-03-31

**Authors:** Wentao Lyu, Xiuting Liu, Lizhi Lu, Bing Dai, Wen Wang, Hua Yang, Yingping Xiao

**Affiliations:** ^1^State Key Laboratory for Managing Biotic and Chemical Threats to the Quality and Safety of Agro-Products, Institute of Quality and Standard for Agro-Products, Zhejiang Academy of Agricultural Sciences, Hangzhou, China; ^2^Institute of Animal Husbandry and Veterinary Science, Zhejiang Academy of Agricultural Sciences, Hangzhou, China; ^3^College of Animal Sciences & Technology, Zhejiang A & F University, Hangzhou, China

**Keywords:** abdominal fat deposition, cecal microbiota, muscovy ducks, *Ruminococcus_torques_group*, *Treponema*

## Abstract

Ducks with the same genetic background vary greatly in their adiposity phenotypes. The gut microbiota plays an essential role in host physiological development and metabolism including fat deposition. However, the association of the gut microbiota with the lipogenic phenotype of ducks remains unknown. In this study, we investigated the cecal microbiota of adult Muscovy ducks and the correlation of the cecal microbiota with fat phenotypes. A total of 200 Muscovy ducks were selected from a population of 5,000 Muscovy ducks to record their abdominal fat weight and collect their cecal contents after being slaughtered and defeathered. The cecal contents were subjective to DNA isolation and 16S rRNA gene sequencing. The results were sorted according to the percentage of abdominal fat and the top 20% (*n* = 40) and the bottom 20% (*n* = 40) were set as the high and low groups, respectively. Our results indicated that in the cecum of Muscovy ducks, Bacteroidetes, Firmicutes, and Fusobacteria were the predominant phyla while *Bacteroides, Oscillospiraceae_uncultured, Parabacteroides*, and *Bacteroidales_norank* were the top 4 dominant genera. Abdominal fat weight (18.57~138.10 g) and percentage of abdominal fat (1.02~27.12%) were significantly correlated (*R*^2^ = 0.92, *P* < 0.001). Although the lipogenic phenotypes of ducks had a significant difference (*P* < 0.05), the α-diversities of the high and low groups were not significantly different (*P* > 0.05). Nevertheless, after random forest analysis, we identified two genera, *Treponema* and *Ruminococcus_torques_group*, that were significantly associated with fat deposition in Muscovy ducks. In addition, the abundances of *Treponema* and *Ruminococcus_torques_group* gave a significantly negative and positive association with abdominal fat weight, respectively (*P* < 0.05). Ducks with a low level of *Treponema* exhibited a tendency toward a high percentage of abdominal fat (*P* < 0.01), while the percentage of abdominal fat in ducks with high *Ruminococcus_torques_group* abundance tended to be higher than that in ducks with low *Ruminococcus_torques_group* abundance (*P* < 0.01). These findings could provide the basic data on the cecal microbiota in Muscovy ducks as well as a theoretical foundation to limit the fat deposition by modulating the gut microbiota in the duck industry.

## Introduction

The global prevalence of obesity and obesity-related diseases has been increasing rapidly (1). Duck meat provides another protein source mainly in Asia, with a high content of essential unsaturated fatty acids, and it is popular in China. However, modern rearing conditions may lead to various developmental and metabolic disorders (2). Excessive abdominal fat deposition is a waste of dietary energy, which is more preferentially deposited into edible parts such as muscle (3). Additionally, considering health, people are consciously limiting the intake of lipids, so consumers would prefer duck meat with high intramuscular content instead of abdominal fat (4). In the poultry industry, approximately 3 million tons of abdominal fat are discarded each year worldwide, leading to a $2.7 billion loss (5).

Similar to in humans and other farm animals, trillions of commensal microorganisms are harbored in the duck gastrointestinal tract, functioning to facilitate the digestion and absorption of nutrients and energy from the diet (6, 7). Therefore, it is possible to develop strategies to enhance physiological status, including growth performance, by altering the gut microbiota. Fecal microbiota transplantation experiments, transferring fecal microbiota from obese humans, mice or pigs to germ-free or antibiotic-treated mice, boosted fat deposition (8–10). This finding indicates that the gut microbiota plays a vital role in the fat metabolism. Furthermore, two microbial taxa, *Methanobrevibacter* and *Mucispirillumschaedleri* were identified from broiler cecal content with a significant correlation with fat deposition (5).

Muscovy ducks (*Cairinamoschata*) have been domesticated for centuries and are characterized by strong-tasting meat with leanness and tenderness (11). Muscovy ducks are excellent meat ducks with high nutritional value, exhibiting high content of unsaturated fatty acids, various amino acids, vitamin E, vitamin B, and trace elements such as iron, copper, zinc, etc. Most investigations on Muscovy ducks involve reovirus and parvovirus (12–16). A recent study revealed that variability in fat accumulation was correlated with the cecal microbiota in chickens (5). The authors also found two microbial taxa, *Methanobrevibacter* and *Mucispirillumschaedleri*, with a strong association with fat deposition. A further understanding of the gut microbiota is needed to control the abdominal fat deposition by regulating the gut microbiota. Little is known about the gut microbiota composition in Muscovy ducks. The present study aimed to study the cecal microbial composition in Muscovy ducks and associate the relative contribution of the cecal microbiota to fat-related phenotypes. In the present study, we performed 16S rRNA gene sequencing on cecal content samples from 200 Muscovy ducks to investigate the commensal bacterial composition in the cecum of Muscovy ducks. We found three predominant phyla, Bacteroidetes, Firmicutes, and Fusobacteria, and four most abundant genera, *Bacteroides, Oscillospiraceae_uncultured, Parabacteroides*, and *Bacteroidales_norank*. After random forest analysis, we identified two genera, *Treponema* and *Ruminococcus_torques_group*, that were negatively and positively correlated with fat-related phenotypes, respectively. The findings of this study will provide insights into the association between the microbial community in the cecum of Muscovy ducks and fat-related phenotypes.

## Materials and Methods

### Ducks and Sample Collection

The Muscovy ducks were from Hewang Poultry Industry Co., Ltd. (Lanxi County, Jinhua City, Zhejiang Province, China). A population of 5,000 female ducks hatched on the same day were fed in cage-free pens on the plastic mesh floor with *ad libitum* water and diets under standardized conditions. Ducks received a commercial starter and finisher feed from 1 to 14 days old and 15 to 70 days old, respectively. The starter and finisher diets composition were stated in Table 1 according to previous studies (17, 18). The energy and nutrients levels met the estimated requirements for ducks (19). On day 70, a total of 200 healthy Muscovy ducks were randomly picked and weighed from the population of 5,000 ducks. All the 200 ducks were weighed and euthanized by CO_2_ asphyxiation. After being weighed and slaughtered, the birds were defeathered and opened to record the weight of abdominal fat tissues. The body weight, the eviscerated weight and the weight of the abdominal fat tissue were recorded promptly. The cecal contents were collected, frozen in liquid nitrogen immediately, and stored at −80°C for DNA isolation. The abdominal fat percentage (AFP) was calculated according to the following formula

$$\begin{array}{l} \text{AFP}=\frac{AFW}{AFW+EW}\;\times \;100\% \end{array}$$

where AFW denotes the abdominal fat weight and EW the eviscerated weight (20).

**Table 1:** Composition and nutrient levels of diets.

**Ingredient (%)**	**Starter**	**Finisher**
Corn	58.90	56.50
Soybean meal	28.00	20.00
Wheat	7.27	18.00
Soybean oil	2.05	1.85
Sodium carbonate	1.14	1.16
Dicalcium phosphate	0.68	0.64
Lysine	0.285	0.315
Methionine	0.265	0.235
NaCl	0.40	0.24
Choline chloride	0.06	0.06
Vitamin and trace mineral premix^a^	1.00	1.00
**Calculated nutrients levels (%)**		
Metabolizable energy, MJ/kg	12.12	11.58
Crude protein	20.50	16.50
Calcium	0.86	0.95
Phosphorus	0.53	0.52
Lysine	0.89	0.92
Methionine	0.51	0.49

a *The premix provided per kilogram of total diet: vitamin A, 10,000 IU; vitamin D3, 2100 IU; vitamin E, 15 IU; vitamin K3, 1 mg; vitamin B1, 2 mg; vitamin B2, 4 mg; vitamin B6, 3 mg; vitamin B12, 0.005 mg; nicotinic acid, 40 mg; pantothenic acid, 10 mg; folic acid, l mg; biotin, 0.3 mg; choline, 2,000 mg; Fe, 120 mg; Cu, 5 mg; Mn, 60 mg; Zn, 25 g; I, 0.3 mg; Se, 0.2 mg*.

Eighty ducks with highest and lowest AFP were selected as high and low groups, respectively.

### DNA Extraction and Sequencing

Genomic DNA was isolated from each cecal sample by a QIAamp DNA Stool Mini Kit (Qiagen, Valencia, CA) according to the manufacturers' instructions. The quality and concentration were evaluated using 1% agarose gel electrophoresis and a NanoDrop ND-1000 (Thermo Fisher Scientific, Waltham, MA, USA). High quality DNA was sequenced by next-generation sequencing (21). In detail, the barcode-fusion forward primer 515F (5′-GTGCCAGCMGCCGCGGTAA-3′) and the reverse primer 907R (5′- CCGTCAATTCMTTTRAGTTT-3′) were employed to amplify the V4–V5 region of the bacterial 16S rRNA gene. The PCR conditions were as previously described (10). After PCR, amplicons were separated and qualified by using 2% (w/v) agarose gels. The DNA samples with 400–450 bp bright bands between were chosen for further experiments. The DNA products were then purified by a GeneJET Gel Extraction Kit (Thermo Scientific) according to the manufacturer's instructions. An Illumina TruSeq DNA PCR-Free Library Preparation Kit (Illumina) was applied for sequencing library generation. The quality of the generated library was evaluated by using a Qubit 2.0 Fluorometer (Thermo Scientific) and an Agilent Bioanalyzer 2100 system. The qualified library was sequenced commercially by Mingke Biotechnology (Hangzhou) on an Illumina HiSeq platform, generating 250 bp paired-end read.

### Bioinformatics Analysis

Illumina paired-end reads were demultiplexed and filtered in Quantitative Insights into Microbial Ecology (QIIME) quality filters (22) for clean reads, merged into tags using FLASH (23), and assigned to each sample in accordance with the unique barcode. The tags of each sample were analyzed after removing redundancies, and unique tags with ≥97% sequence similarity were assigned to the same operational taxonomic units (OTUs) using UPARSE and UCHIME. Selected OTUs were annotated with taxonomic information using the RDP classifier (24). Alpha-diversity (Observed Species, Chao 1 estimator, ACE, Shannon, and Simpson indices) and beta-diversity were calculated and visualized in OriginLab 2018 (Northampton, MA, US).

### Identification of Adipogenesis-Related Microbiota

To detect the microorganisms significantly related with fat deposition, the taxa present in <30% of samples in Muscovy duck cecum were excluded. All the samples were successively sorted by host BW, AFW, and AFP and the relative abundance of each microorganism. The lowest 20% and highest 20% of the ranked birds were considered two distinct groups, and statistical analysis was performed for all the traits between the two groups. Furthermore, regression-based random forest models were performed to identify bacterial features associated with fat deposition in the randomForest package in the R project (25). Subsequently, the BW, AFW, and AFP were statically analyzed between the lowest 20% and highest 20% relative abundance of the common bacteria in fat-related bacterial characterization (detected in at least 30% of the cecal samples).

### Statistical Analysis

Data are expressed as the mean ± SEM. All statistical analyses were performed in OriginLab 2018. The difference between two groups was analyzed by unpaired two-tailed Student's *t*-test and considered significant when the *P*-value was no more than 0.05.

### Accession Number

The raw sequencing reads of this study have been deposited in NCBI under the accession number BioProject PRJNA663038.

## Results

### The Composition of the Cecal Microbiota in Muscovy Ducks

**Figure 1:**
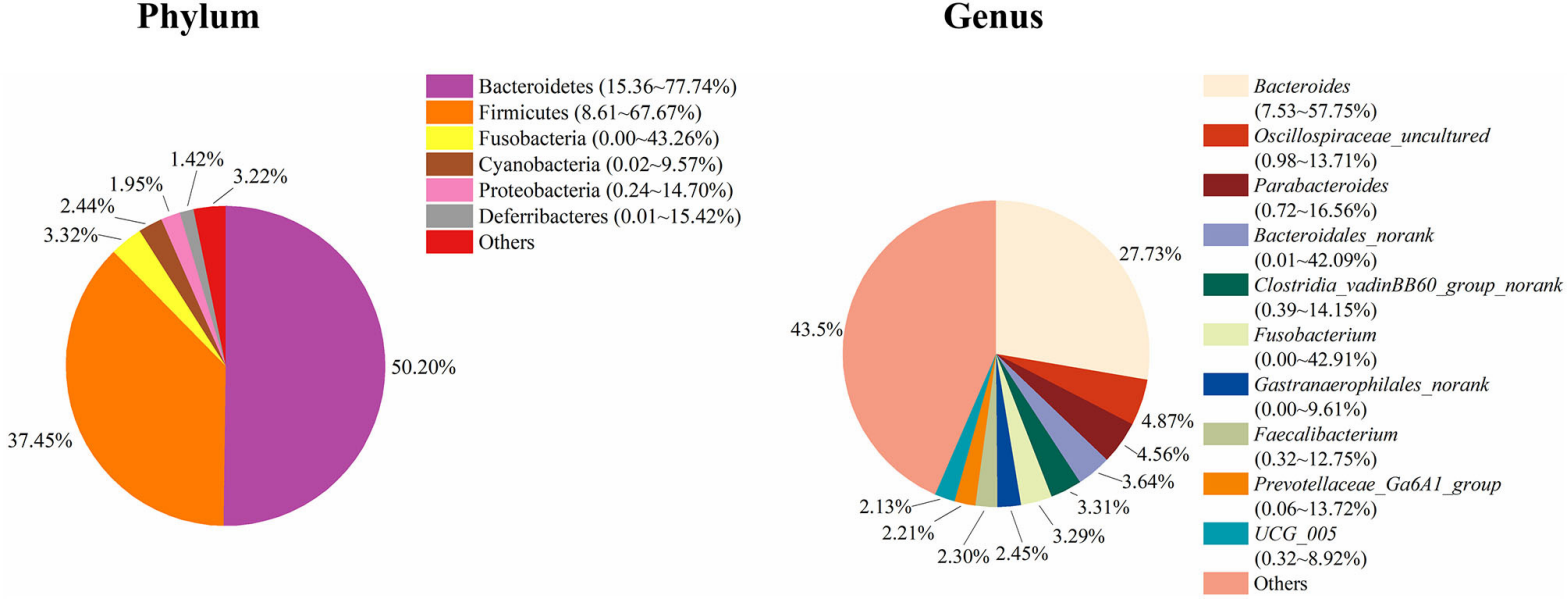
The composition of the cecal microbiota in Muscovy ducks at the phylum and genus levels.

To study the role of the cecal microbiota in adipose accumulation, we first investigated the composition of the cecal microbiota in Muscovy ducks. Cecal content samples from 200 Muscovy ducks were collected for bacterial 16S rRNA gene sequencing. A total of 7,469,466 high-quality sequence tags were generated and classified into 1,989 OTUs at the 97% similarity level. At the phylum level, Bacteroidetes, Firmicutes, Fusobacteria, Cyanobacteria, Proteobacteria, and Deferribacteres were the top six phyla in the cecal contents, accounting for 96.78% of the total abundance (Figure 1). In detail, Bacteroidetes was the predominant phylum and accounted for 50.20% of the total sequences in the cecal content samples of Muscovy ducks, followed by Firmicutes, accounting for 37.45% of the total abundance. At the genus level, due to the richness of the microbiota, we listed only the top 10 genera in the cecal content of Muscovy ducks (Figure 1). Furthermore, the top four genera were *Bacteroides, Oscillospiraceae_uncultured, Parabacteroides*, and *Bacteroidales_norank*, accounting for 40.80% of the total bacteria in the cecum of Muscovy ducks.

### Duck Phenotype Characterization

**Figure 2:**
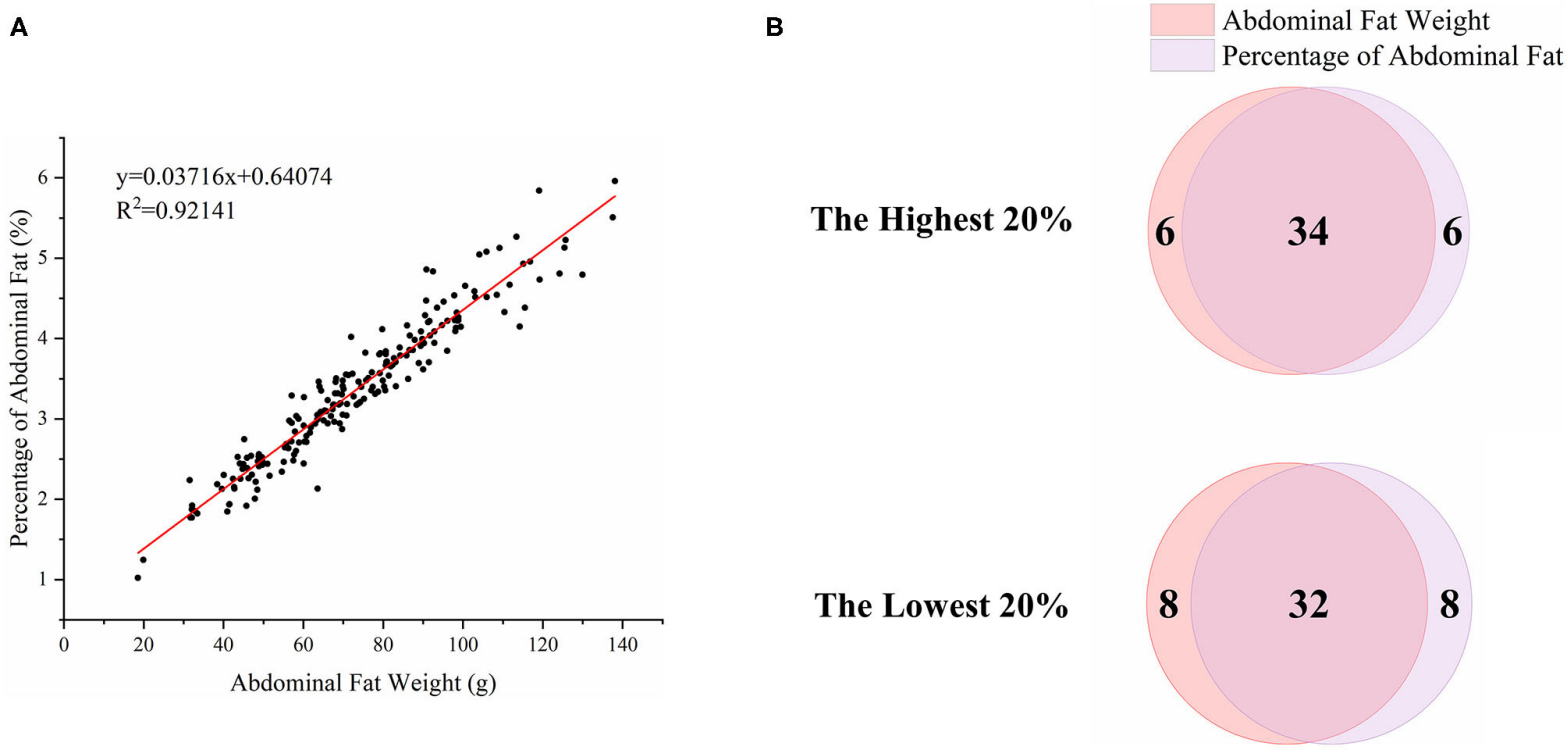
Association analysis of abdominal fat weight (AFW) and percentage of abdominal fat (AFP). **(A)**. The correlation between AFW and AFP. **(B)**. Overlap analysis of the shared birds in both AFW- and AFP-ranked groups of the highest 20% (*n* = 40) and lowest 20% (*n* = 40).

To characterize the distinct phenotype related to fat accumulation, we performed correlation analysis on the three phenotypes, namely, BW, AFW, and AFP. The observations on BW, AFW, and AFP are summarized in Table 2. AFW and AFP gave a high phenotypic correlation (*R*^2^ = 0.92, *P* < 0.001, Figure 2A). However, the *R*^2^ for the correlation between BW and AFW was 0.37 while the *R*^2^ for the correlation between BW and AFP was 0.16.

**Table 2:** Descriptive statistics for Muscovy duck phenotypes.

**Traits**	***N***	**Mean**	**SD**	**CV (%)**	**Maximum**	**Minimum**
BW (kg)	200	2.80	0.26	9.12	3.58	1.97
AFW (g)	200	73.12	23.53	32.18	138.10	18.57
AFP (%)	200	3.36	0.91	27.12	5.96	1.02

*N, the number of non-missing values; SD, standard deviation; CV, coefficient of variation; BW, body weight; AFW, abdominal fat weight; AFP, percentage of abdominal fat*.

**Figure 3:**
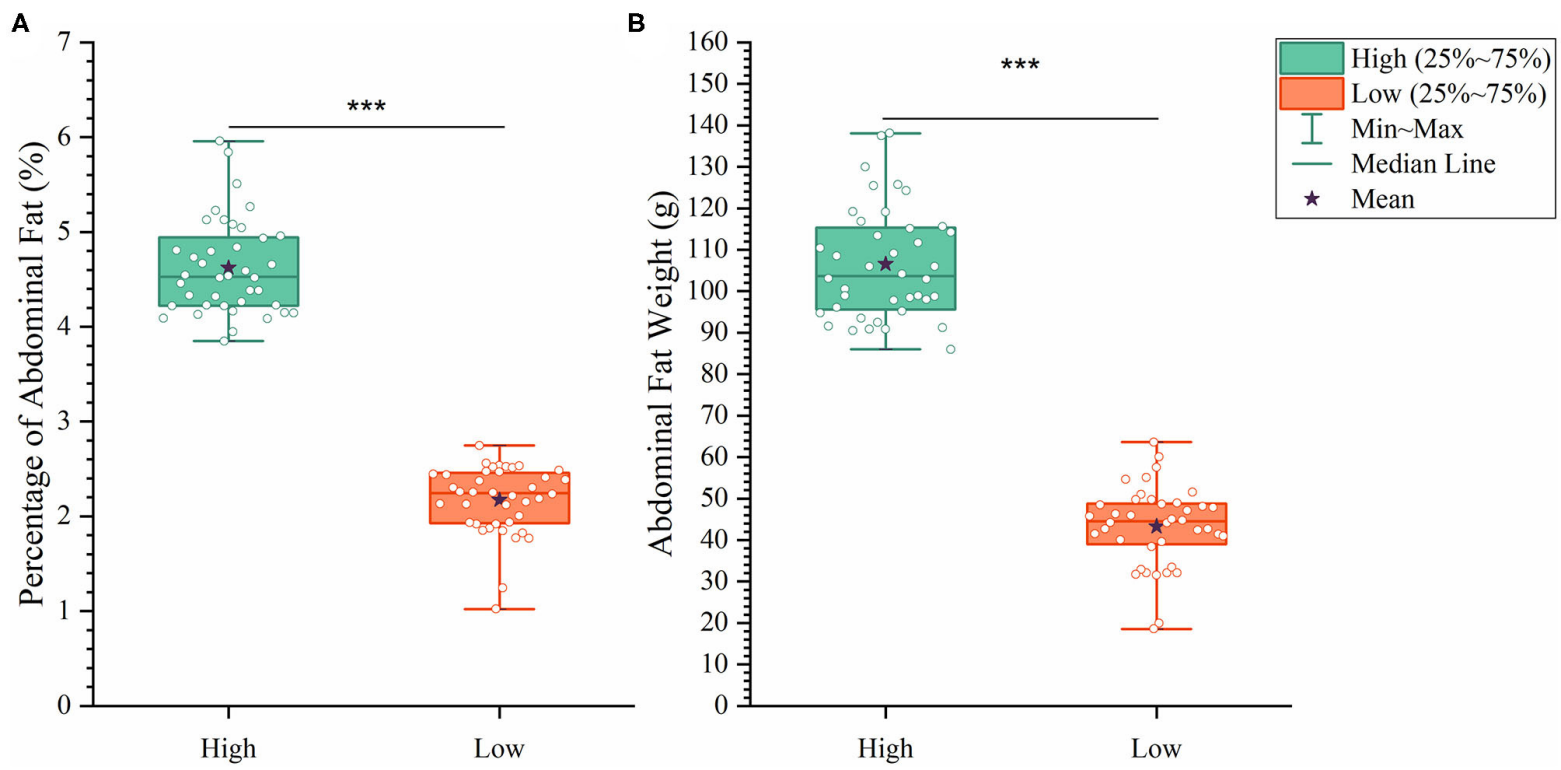
The differences between the high and low groups in percentage of abdominal fat **(A)** and abdominal fat weight **(B)**. ***P < 0.001.

To analyze how AFW and AFP were associated with each other, we compared ducks between the top and bottom 20% birds in terms of AFW and AFP. The 40 ducks with the highest and lowest AFW and AFP shared 34 and 32 individual birds, respectively, because of the high correlation between AFW and AFP (Figure 2B). Consequently, we set the highest and lowest 20% ducks in terms of AFP as the high and low groups, respectively. As shown in Figure 2, all of the AFP, AFW, and BW values gave highly significant differences between Muscovy ducks with the highest and lowest 20% AFP (*n* = 40), suggesting the correlation of AFP with AFW and BW. To visualize the difference between the groups, we sorted the trait results by AFP and set the highest 20% (*n* = 40) and the lowest 20% (*n* = 40) as the high and low groups, respectively. As expected, with a significant difference (*P* < 0.001) in AFP, the AFW of ducks in the high and low groups showed a significant difference (*P* < 0.001, Figure 3).

### Differences in the Diversity, Composition and Potential Function of the Cecal Microbiota

**Figure 4:**
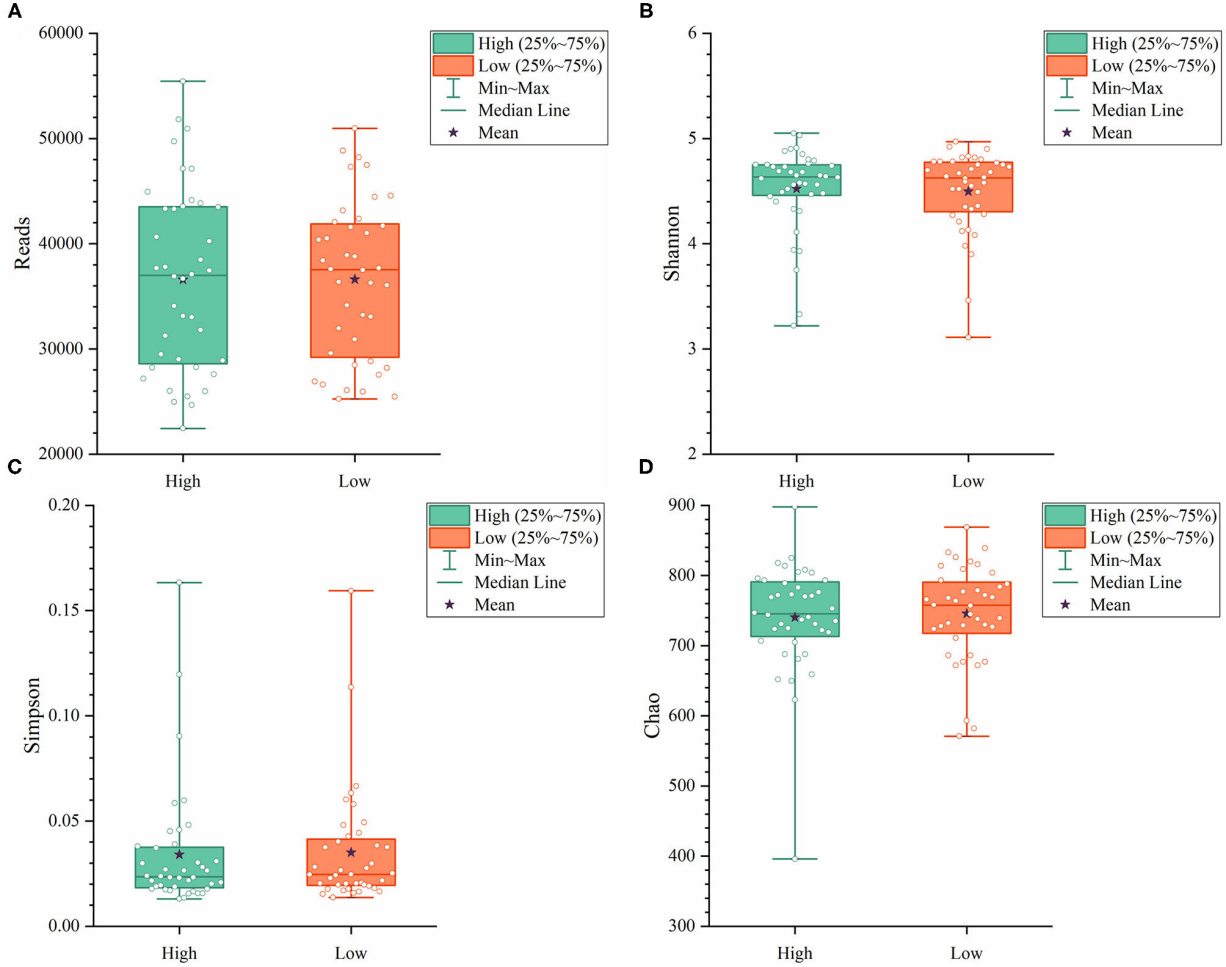
The α-diversity including Reads **(A)**, Shannon **(B)**, Simpson **(C)**, and Chao **(D)** in the cecum of high and low group ducks.

To further explore the differences in the α-diversity and microbial composition between the high and low group Muscovy ducks, we compared the α-diversity and bacterial composition in the cecum of the high and low group ducks. The α-diversity of the cecal microbiota was measured by the Shannon index (Figure 4A), the Simpson index (Figure 4B), and the Chao index (Figure 4C). Overall, there was no significant difference in α-diversity between the high and low groups (*P* > 0.05), suggestive of no significant difference in the microbial richness of the duck cecum in the high and low groups.

**Figure 5:**
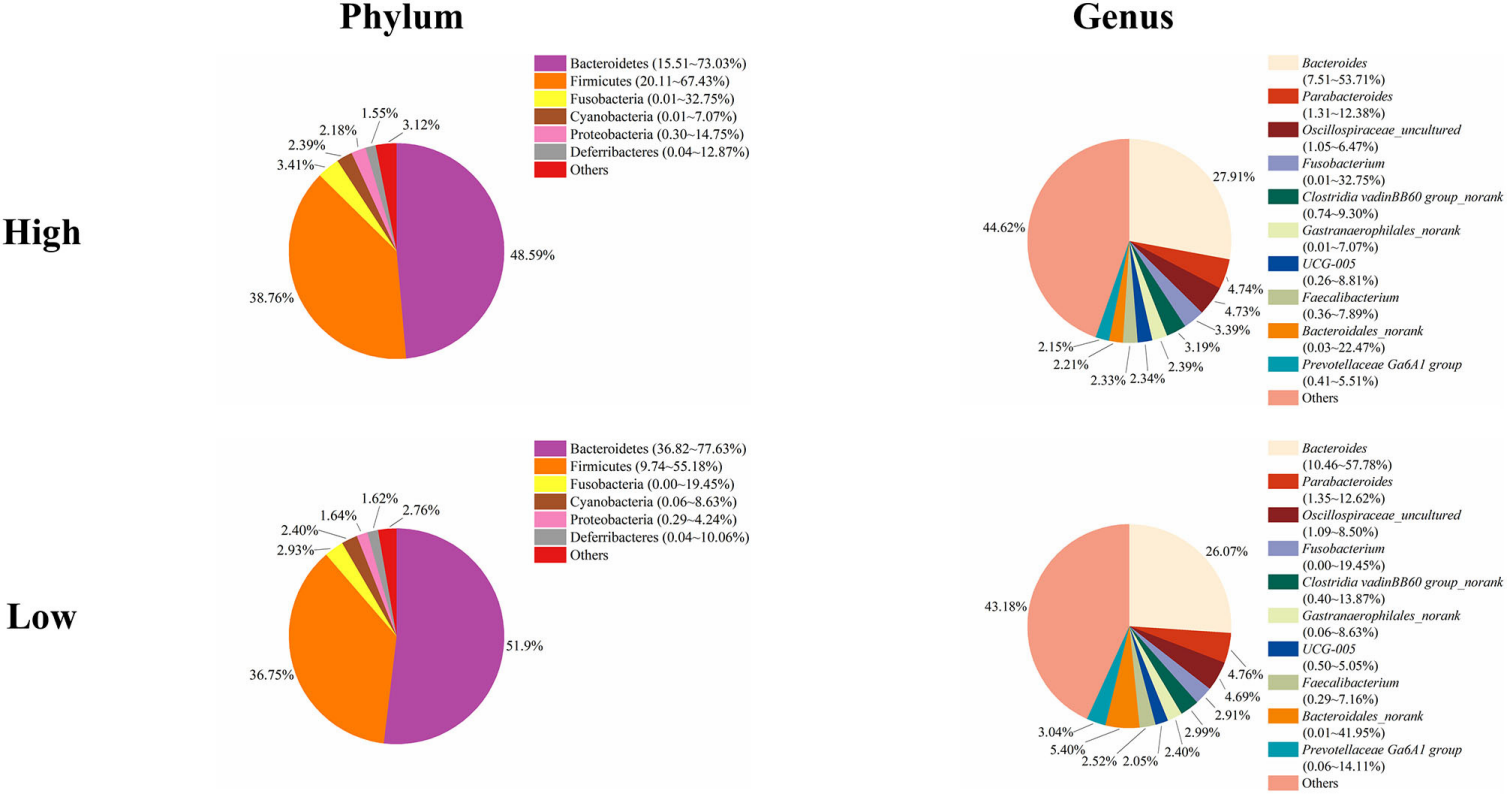
The microbial composition in the cecum of the high and low group ducks.

Next, we analyzed the microbial composition in the cecum of ducks in the high and low groups. The top six phyla in the cecal content of ducks in both the high and low groups were the same as those in the total group of 200 ducks, namely, Bacteroidetes, Firmicutes, Fusobacteria, Cyanobacteria, Proteobacteria, and Deferribacteres with different relative abundances (Figure 5). The cecum of ducks in the high group had a relatively higher abundance of Firmicutes but a lower abundance of Bacteroidetes, accounting for 38.76 and 48.59%, respectively. The relative abundances of the other four genera, Fusobacteria, Cyanobacteria, Proteobacteria, and Deferribacteres, were similar in the cecal samples in the high and low groups. At the genus level, *Bacteroides, Parabacteroides, Oscillospiraceae_uncultured, Fusobacterium, Clostridia vadinBB60 group_norank, Gastranaerophilales_norank, UCG-005, Faecalibacterium, Bacteroidales_norank*, and *Prevotellaceae Ga6A1 group* composed the top 10 genera in both groups of ducks (Figure 5). Among them, *Bacteroides* was slightly less abundant in the low group ducks (27.91%) than in the high group ducks (26.07%). However, the relative abundance of *Bacteroidales* was higher in the low group ducks (5.40%) than in the high group ducks (2.21%). The proportions of the other genera were similar between the high and low group ducks.

### The Genera *Treponema* and *Ruminococcus_torques_group* are Significantly Associated with Fat Deposition

**Figure 6:**
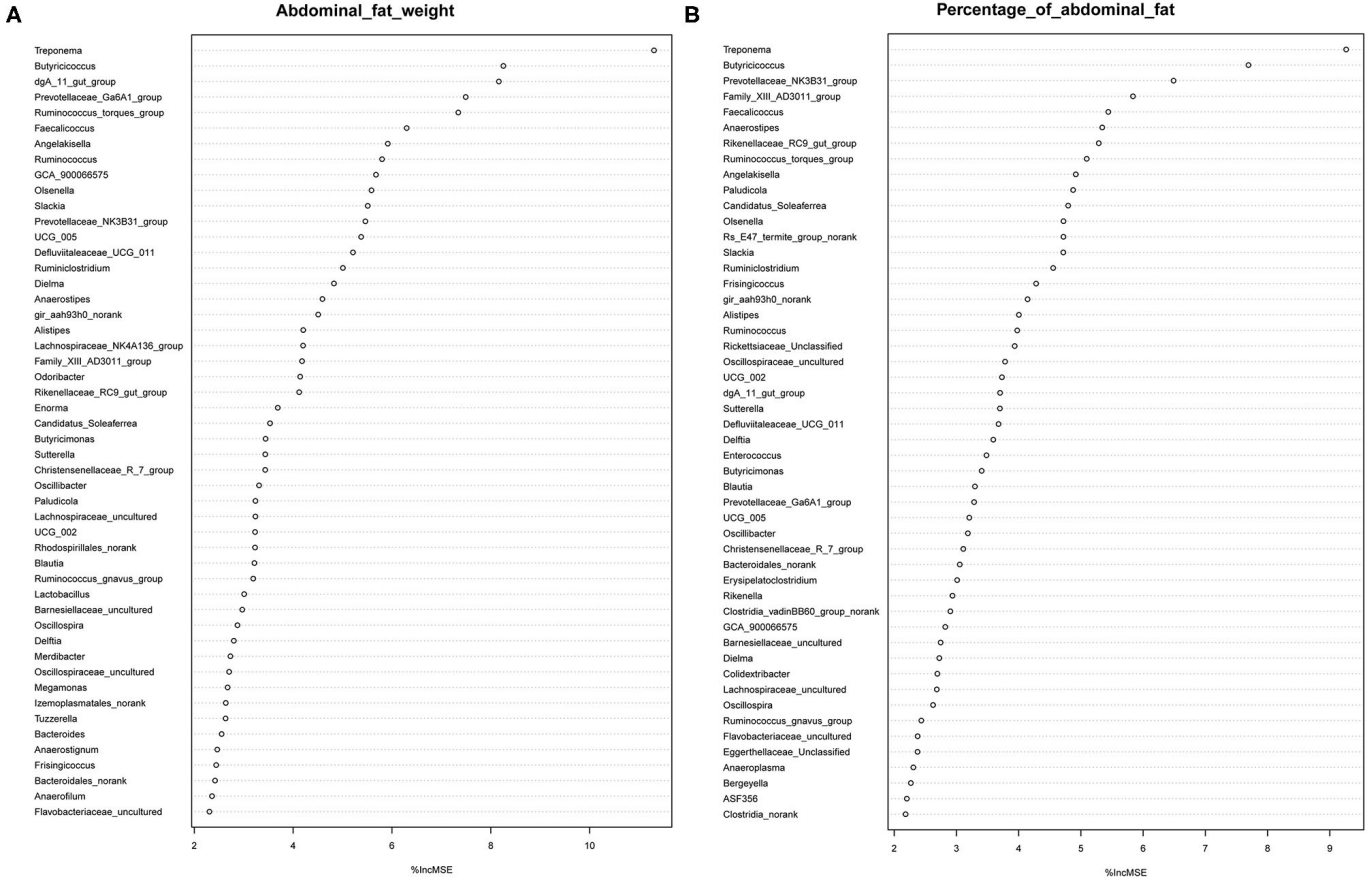
Abdominal fat weight **(A)** and percentage of abdominal fat **(B)** related genus in the cecum of Muscovy ducks.

**Figure 7:**
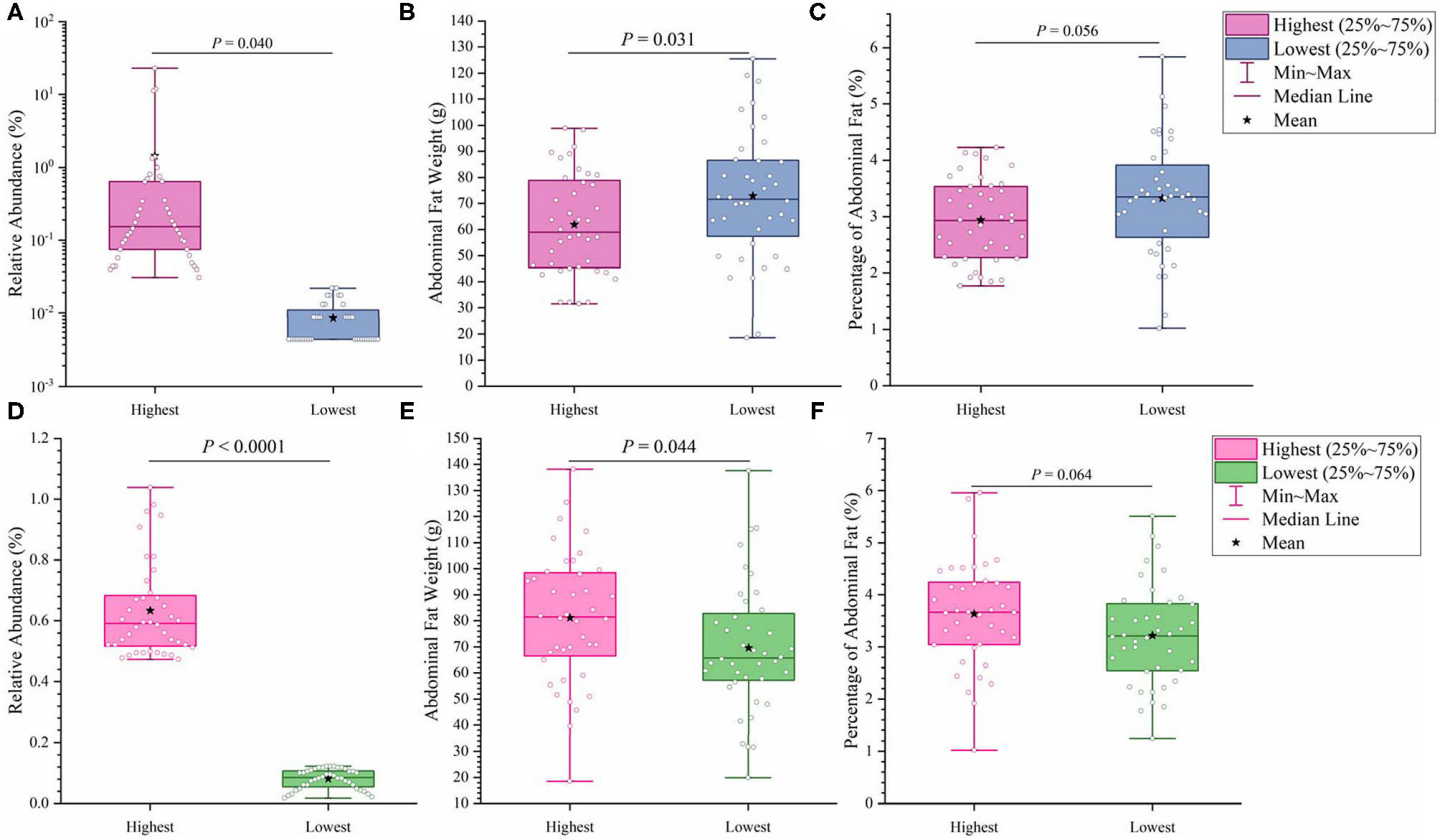
Effects of fatness-related microbial genera, *Treponema* **(A–C)** and *Ruminococcus_torques_group* **(D–F)**, on host phenotypes.

It has previously been suggested that abdominal fat accumulation is associated with the gut microbiota (5). We further investigated which specific bacteria were linked with abdominal fat accumulation by running the randomForest analysis in the R project. The top 10 bacterial features predicting abdominal fat accumulation are listed in Figure 6. The common features in the lists of the top 10 AFW-related and AFP-related features were *Treponema, Butyricicoccus, Ruminococcus_torques_group, Faecalicoccus*, and *Angelakisella*. After adjusting the *P*-value to *P* < 0.05, we focused on the two cecal genera, *Treponema* and *Ruminococcus_torques_group*, to further investigate how these two genera affected abdominal fat accumulation in Muscovy ducks. The ducks with *Treponema* and *Ruminococcus_torques_group* were sorted according to the relative abundances of these two genera. The 40 ducks with the most (20%) and least (20%) abundance of *Treponema* were selected for comparing the AFW, AFP, and BW. The same analysis was performed for *Ruminococcus_torques_group*. As shown in Figure 7, *Treponema* and *Ruminococcus_torques_group* abundances were significantly related to AFW (*P* < 0.05). Furthermore, the 20% of ducks with the highest *Treponema* abundance tended to have a significantly lower AFP and BW than the 20% of ducks with the lowest *Treponema* abundance (Figure 7A, *P* = 0.056 and 0.076), indicating a negative correlation between fat accumulation and *Treponema* abundance. However, the opposite was true with the bacterial genus *Ruminococcus_torques_group*, giving a positive association between fat deposition and the relative abundance of *Ruminococcus_torques_group* (Figure 7B).

## Discussion

Muscovy ducks (*Cairinamoschata*) are characterized by meat quality with low fat and high protein content. The gut microbiota plays an essential role in energy metabolism and fat accumulation in the host and facilitates energy harvest from indigestible dietary fibers via fermentation (8, 27). Besides, the gut microbiota is involved in modulating the host immune system. The components of the gut microbiota vary according to the local microenvironment, such as oxygen gradients, pH levels, and nutrient availability along the gastrointestinal tract (5). Particularly in the cecum, Bacteroidetes and Firmicutes are the three predominant phyla in most animals. 16S rRNA sequencing results showed that the cecal bacteria are mainly composed of the Bacteroidetes and Firmicutes phyla in humans and mice (28), while Bacteroidetes and Firmicutes are the main phyla in the cecal digesta of pigs from farrow to finish (29, 30). Moreover, for chickens, Firmicutes, Bacteroides, Proteobacteria, and Actinobacteria account for more than 99% of the phyla in the cecum, where Firmicutes and Bacteroides are the most common ones (31, 32). Additionally, a study on Shaoxing ducks indicated that the most abundant phyla in the cecum were Firmicutes and Bacteroidetes (33). Not surprisingly, in the present study, we found that Bacteroidetes and Firmicutes were the two predominant phyla, while *Bacteroides* and *Parabacteroides* were the two most abundant genera in the cecum of Muscovy ducks (Figure 1). Due to the cecum being the distal portion of the GI tract, oxygen is limited, and indigestible dietary substrates are fermented in the cecum. Therefore, the bacterial components in the cecum are quite different from those in the small intestine. Due to fermentation mainly taking place in cecum, microbial cells are most abundant in poultry cecum (34). The differences in bacterial component of duck cecum might explain a large portion of microbial regulating fat deposition, that's why we focus on the cecum in the present study. Furthermore, the majority of short chain fatty acids (SCFAs) are produced by bacteria in the cecum from indigestible substrate fermentation as the energy source for host cells as well as microorganisms in the cecum. With deepening microbiota investigations, increasing attention has been paid to the association between the gut microbiota and host energy metabolism and abdominal fat accumulation. The richness of these two phyla is considered to be correlated with the energy absorption from food by the host (8, 35). In the present study, compared to ducks in the low group, ducks in the high group gave a higher relative abundance of the Firmicutes phylum and a lower relative abundance of the Bacteroidetes phylum (Figure 5).

Among the top 10 AFW-related and AFP-related genera, *Treponema* and *Ruminococcus_torques_group* were found to be negatively and positively associated with fat deposition, respectively. With the ability to facilitate the absorption of energy from dietary indigestible polysaccharides (36), the abundance of *Treponema* groups is known to be negatively associated with fat content in the diet. With 99% identity of *Treponema* spp. with *T. succinifaciens, Treponema* spp. might contribute to host energy metabolism because *T. succinifaciens* is a carbohydrate metabolizer in the gut of termites, swine, and cattle (37). After the analysis of the gut microbiome in Japanese macaques, Prince et al. (38) found that long exposure to a high fat diet was strongly correlated with low *Treponema* abundance, suggestive of an association between high dietary fat content and low *Treponema* abundance. This result was confirmed by De Filippo et al. (39) who found that the *Treponema* abundance in leaner children from rural village (BR) and Nanoro town (BT) was significantly higher than that in children from the capital city of Burkina Faso (BC) and Europe (EU). Additionally, from 300 porcine cecum lumen samples, association analysis showed that *Treponema* was one of the most negatively intramuscular fat-associated bacteria (40), providing additional evidence that *Treponema* is involved in fat deposition with a negative correlation. Consistently, we observed that the high *Treponema* abundance group had a significantly lower AFW than the low *Treponema* abundance group (*P* < 0.05). The AFP of ducks in the high *Treponema* abundance group tended be lower than that of ducks in the low *Treponema* abundance group (*P* < 0.01; Figure 7).

*Ruminococcus_torques_group*is a genera derived from genus *Mediterraneibacter* from the family Lachnospiraceae (NCBI Taxonomy Browser, https://www.ncbi.nlm.nih.gov/Taxonomy/Browser/wwwtax.cgi) and was discovered to ferment gastric mucins and identified as a butyrate-producing bacterium (41). A study on Fubrick tea aqueous extract (FTEs) in mice indicated that FTEs were able to alleviate visceral fat deposition via an increase in the abundance of Lachnospiraceae (42). This result suggests a positive correlation between fat deposition and Lachnospiraceae abundance. A similar positive association of abdominal fat and subcutaneous fat thickness with Lachnospiraceae abundance was observed in broilers (43). Because it is a species of the family Lachnospiraceae, the abundance of *Ruminococcus_torques_group* is supposed to be positively related to fat accumulation. Besides, a randomized, double-blinded and crossover design clinical trial on resistant starch showed that a low level of *Ruminococcus_torques_group* would assist in reducing the body fat (44), suggestive of a positive relationship between the *Ruminococcus_torques_group* level and fat accumulation. Additionally, *Ruminococcus_torques_group*, categorized as *Clostridium* cluster XIVa, mainly produces butyrate (45). Butyrate-producing bacteria were found to promote fat deposition (46) because they are able to convert dietary fiber to butyrate by fermentation to provide additional energy to the host (26). In our study, the ducks with high *Ruminococcus_torques_group* abundance showed significantly higher AFW (*P* < 0.05) and a tendency toward higher AFP (*P* < 0.01) than the ducks with low *Ruminococcus_torques_group* abundance (Figure 7B). No significant difference was observed in BW between the high and low *Ruminococcus_torques_group* abundance groups. Our result and previous studies illustrate the positive correlation of the *Ruminococcus_torques_group* level with fat deposition.

Collectively, we thoroughly investigated the Muscovy duck cecal microbiota by 16S rRNA sequencing and analyzed the correlation of duck fat deposition with the cecal microbiota. It is not surprising that various bacteria existed in the duck cecum with the dominant phyla being Bacteroidetes and Firmicutes, giving a result similar to that with other duck species. The variability in fat deposition of a duck population was correlated with the cecal microbiota in Muscovy ducks. Interestingly, the genera *Treponema* and *Ruminococcus_torques_group* were negatively and positively associated with fat deposition, respectively. These results would provide basic evidence that the cecal microbiota contributes to regulating fat deposition and is beneficial to the development of strategies for abdominal fat reduction in the duck industry.

## Data Availability Statement

The datasets generated for this study can be found in online repositories. The names of the repository/repositories and accession number(s) can be found below: https://www.ncbi.nlm.nih.gov/, BioProject PRJNA663038.

## Ethics Statement

The animal study was reviewed and approved by Animal Care and Use Committee of Zhejiang Academy of Agricultural Sciences.

## Author Contributions

WL, XL, LL, BD, and WW: conceptualization, methodology, and experiment performance. WL: data analysis, writing—original draft preparation. HY and YX: writing—review and editing. All authors have read and agreed to the published version of the manuscript.

## Conflict of Interest

The authors declare that the research was conducted in the absence of any commercial or financial relationships that could be construed as a potential conflict of interest.
